# Generation of Variability in *Chrysodeixis includens* Nucleopolyhedrovirus (ChinNPV): The Role of a Single Variant

**DOI:** 10.3390/v13101895

**Published:** 2021-09-22

**Authors:** Eduardo Aguirre, Inés Beperet, Trevor Williams, Primitivo Caballero

**Affiliations:** 1 Institute for Multidisciplinary Research in Applied Biology, Universidad Pública de Navarra, 31006 Pamplona, Spain; eduardo.aguirre@unavarra.es; 2 Departamento de Investigación y Desarrollo, Bioinsectis SL, 31110 Noain, Spain; ines.beperet@bioinsectis.com; 3 Instituto de Ecología AC, Xalapa 91073, Mexico

**Keywords:** ChinNPV, variability, prevalence, SNPs, concentration, *Chrysodeixis includens*

## Abstract

The mechanisms generating variability in viruses are diverse. Variability allows baculoviruses to evolve with their host and with changes in their environment. We examined the role of one genetic variant of Chrysodeixis includens nucleopolyhedrovirus (ChinNPV) and its contribution to the variability of the virus under laboratory conditions. A mixture of natural isolates (ChinNPV-Mex1) contained two genetic variants that dominated over other variants in individual larvae that consumed high (ChinNPV-K) and low (ChinNPV-E) concentrations of inoculum. Studies on the ChinNPV-K variant indicated that it was capable of generating novel variation in a concentration-dependent manner. In cell culture, cells inoculated with high concentrations of ChinNPV-K produced OBs with the ChinNPV-K REN profile, whereas a high diversity of ChinNPV variants was recovered following plaque purification of low concentrations of ChinNPV-K virion inoculum. Interestingly, the ChinNPV-K variant could not be recovered from plaques derived from low concentration inocula originating from budded virions or occlusion-derived virions of ChinNPV-K. Genome sequencing revealed marked differences between ChinNPV-K and ChinNPV-E, with high variation in the ChinNPV-K genome, mostly due to single nucleotide polymorphisms. We conclude that ChinNPV-K is an unstable genetic variant that is responsible for generating much of the detected variability in the natural ChinNPV isolates used in this study.

## 1. Introduction

Baculoviruses are double-stranded DNA viruses, with a large circular genome ranging from 80 to 180 Kb [1]. Due to the negative correlation between mutation rate and genome length, baculoviruses are supposed to be less prone to mutation than most other DNA viruses [2]. Nevertheless, natural isolates of baculoviruses are known to comprise mixtures of genotypes [3], which suggests that phenomena such as recombination, mutation, and transposition take place frequently during baculovirus replication [4,5,6,7,8]. In addition, single nucleotide polymorphisms (SNPs) can be generated during the replication of viral DNA. SNPs could arise from a lack of proof reading activity of the viral polymerase, although polymerases from Bombyx mori NPV (BmNPV) and Autographa californica MNPV (AcMNPV) both present 3′ to 5′ exonuclease activity, which implies a high degree of fidelity during the DNA replication cycle [9,10,11].

The presence of high diversity within baculoviruses is particularly evident when sequencing these viruses. Two recent examples, a study carried out on Spodoptera frugiperda multiple nucleopolyhedrovirus (SfMNPV) [12] and another on Autographa californica multiple nucleopolyhedrovirus (AcMNPV) [13], revealed the high variability present in natural baculovirus isolates and the mechanisms likely to be involved in its generation. Factors such as food plants or host genetic heterogeneity can also directly influence the genetic variability present in baculovirus isolates [14,15]. A previous study in *Helicoverpa armigera* identified the role of the inoculum dose on the genetic variability present in an isolate of Helicoverpa armigera NPV (HearNPV) [16]. In that study, the authors examined the positive effect of inoculum dose in the variability observed when *H. armigera* larvae were fed with 5% or 95% lethal doses (LD_5_ or LD_95_) of HearNPV. The variability observed was similar in insects that consumed either of the two doses, which was unexpected given that the authors hypothesized that the genetic diversity detected in the lower dose would be lower than the genetic diversity of the samples used for inoculation [16].

High genetic diversity is present in isolates of Chrysodeixis includens nucleopolyhedrovirus (ChinNPV, previously known as Pseudoplusia includens single nucleopolyhedrovirus) collected in soybean fields in Mexico [17]. Nine genetically distinct isolates were obtained from eleven virus-killed larvae. In vitro cloning from an equimolar mixture of the nine isolates, named ChinNPV-Mex1, revealed the presence of 23 genetic variants by restriction endonuclease analysis [17]. Here, we examined the effect of host heterogeneity and inoculum concentration on the genetic variability of this artificial mixture. We also reveal the key role of a single variant, named ChinNPV-K, in generating variability in a concentration-dependent manner. This variation was mainly present as SNPs throughout the genome. The molecular mechanisms that generate this variability are unclear, although several possibilities are discussed.

## 2. Materials and Methods

### 2.1. Insects, Cells, and Viruses

A laboratory colony of *C. includens* was established from larvae collected in soybean fields of Tamaulipas, Mexico, and maintained under controlled conditions at 25 ± 1 °C, 75% relative humidity (RH) and 16 h light: 8 h dark photoperiod. Larvae were reared on a wheatgerm-based semi-synthetic diet [18]. This population was tested by qPCR following the method described by Virto et al. [19] and declared as ChinNPV-free (data not shown). HighFive cells from *Trichoplusia ni* (ThermoFisher Scientific, Waltham, MA, USA) were maintained in TNM-FH medium (Gibco, Life Technologies Ltd., Renfrew, UK) with 10% fetal bovine serum (Gibco, Life Technologies Ltd., Renfrew, UK) at 28 °C.

ChinNPV-Mex1 was prepared from an equimolar mixture of viral occlusion bodies (OBs) from nine ChinNPV field isolates described previously [17]. Individual genotypes used in this study were isolated from ChinNPV-Mex1 by plaque purification in HighFive cells, multiplied in *C. includens* fifth instars and identified as distinct variants using the restriction endonucleases EcoRI and HindIII [17]. To purify OBs, each virus-killed larva was filtered through muslin and centrifuged with 0.1% SDS twice at 2300× *g* to eliminate insect debris. The resulting pellets were washed in distilled water and resuspended in milli-Q water. OB concentrations were determined by counting in an improved hemocytometer (Hawksley Ltd., Lancing, UK) under phase-contrast microscopy. Purified OBs were stored at 4 °C until required.

### 2.2. Viral DNA Extraction and Restriction Endonuclease Analysis (REN)

Occlusion-derived virions (ODVs) were released from OBs by incubating 20 μL of OB suspension (~10^10^ OBs/mL) with 50 μL of 0.5 M Na_2_CO_3_ and 180 μL of distilled water at 60 °C for 10 min. The suspension was centrifuged at 6000× *g* for 5 min and the supernatant containing the virions was incubated for 1 h at 65 °C with 15 μL proteinase K (20 mg/mL), 25 μL of 10% SDS and 25 μL of 0.5 M EDTA. Viral DNA was then separated from proteins by the addition of 150 μL of MPC reagent (Masterpure™ Kit, Lucigen Corp., Middleton, WI, USA) and centrifuged for 10 min at 10,000× *g*. DNA was precipitated using 2.5 volumes of ice cold absolute ethanol for 10 min at 12,000× *g*. Pelleted DNA was washed twice with 70% ice-cold ethanol and resuspended in 50 μL 0.1 × TE buffer (10 mM Tris, 1 mM EDTA pH 8.0). The final DNA concentration was estimated using a Nanodrop One (ThermoFisher Scientific, Waltham, MA, USA) spectrophotometer.

For restriction endonuclease (REN) analysis, 2 μg of viral DNA of each ChinNPV genotype were digested with EcoRI and HindIII (FastDigest™, Thermo Fisher Scientific Inc., Waltham, MA, USA) for 1 h at 37 °C. The reactions were loaded into a 1% agarose gel and fragments were then separated by electrophoresis in 1 × TAE buffer (40 mM Tris, 20 mM acetic acid, 1 mM EDTA pH 8.0) running at 18 V for 15 h. DNA was stained with GelRed^®^ (Biotium Inc., Fremont, CA, USA) and visualized on a UV transilluminator (G-Box Syngene, Synoptics Ltd., Cambridge, UK).

### 2.3. Effects of Host Heterogeneity and Inoculum Concentration on Genetic Diversity Following Inoculation of Larvae with ChinNPV-Mex1

To examine the effects of host heterogeneity and inoculum concentration on variant diversity, individual fifth instar *C. includens* larvae were inoculated with one of four different OB concentrations: 1 × 10^4^, 5 × 10^5^, 5 × 10^6^ and 1 × 10^8^ OBs/mL, using the droplet feeding method [20]. This range of OB concentrations was expected to result in between 20% and 100% mortality, based on the results of previous assays. Larvae were inoculated with each OB concentration and each larva was considered as a replicate insect. A total of 447 larvae were inoculated for the 1 × 10^4^ OBs/mL concentration, 72 larvae for the 5 × 10^5^ OBs/mL concentration, 150 larvae for the 5 × 10^6^ OBs/mL concentration and 100 larvae for the 10^8^ OBs/mL concentration. These treatments were applied using eight different batches of larvae (~100 larvae/batch) to account for variation in host susceptibility to infection. Differences in the numbers of inoculated larvae for each concentration reflected the need of achieve a minimum of 60–100 virus-killed individuals for each concentration. Larvae were reared individually on semi-synthetic diet and incubated in 16 h light: 8 h dark photoperiod at 25 ± 1 °C and 75% relative humidity. Virus induced mortality was recorded every 24 h until larvae had died or pupated. Control larvae (20 individuals) drank a solution of sucrose and food dye containing no OBs. All virus-killed larvae were recovered individually, and the OBs purified as described in Section 2.1. REN analysis was performed on the DNA extracted from the recovered OBs using EcoRI and HindIII. The most abundant genetic variant present in each insect was identified by comparing each REN profile with the REN profiles of the genotypes previously isolated from the ChinNPV-Mex1 mixture [17]. New REN profiles that did not correspond to the genetic variants identified in the previous study were classified together in the same group as “unidentified” variants. The prevalence of each dominant genotype was expressed as the percentage of larvae that produced OBs with a particular REN profile. The effect of OB concentration on the frequency of ChinNPV-K REN profiles was examined using Pearson’s χ^2^ test in SPSS v25.0 software [21].

### 2.4. Genetic Diversity Present Following Inoculation with Variants ChinNPV-K and ChinNPV-E

The ChinNPV-E and -K variants were selected for study because their characteristic REN profiles were the most prevalent in insects inoculated with low and high concentrations of OBs, respectively, in the previous experiment (Section 2.3). A similar experiment to that of Section 2.3 was performed using OBs comprising ChinNPV-E and -K variants alone. To simplify the experiment, we used only two OB concentrations (5 × 10^4^ and 1 × 10^8^ OBs/mL), estimated to result in approximately 20% and 99% mortality, respectively. A total of 145–163 larvae were inoculated with the 1 × 10^8^ OBs/mL concentration, and 169–368 larvae for the 5 × 10^4^ OBs/mL concentration, in order to achieve a total of 144–162 virus-killed larvae for the 1 × 10^8^ OBs/mL and 57–80 virus-killed larvae for the 5 × 10^4^ OBs/mL concentration. As in the previous experiment, treatments were applied to eight different batches of larvae. A group of 20 control larvae were treated identically but did not consume OBs. The results were analyzed as described in Section 2.3. The frequencies of ChinNPV-K or -E virus-killed larvae were compared by Fisher’s exact test with SPSS v25.0 [21].

### 2.5. Genetic Stability of Progeny OBs Obtained Following Infection with ChinNPV-K OBs at Low Concentration

Due to the high variability of genotypes obtained in the ChinNPV-K low concentration treatment in the previous experiment (Section 2.4), we selected four OB samples from the previous experiment that showed the dominant REN profile of genotypic variants ChinNPV-H, -L, -N and -P. Each of these variants had been recovered from the 5 × 10^4^ OBs/mL concentration treatment in Section 2.4. Each OB sample was used as inoculum for another assay involving high inoculum concentration to determine whether the ChinNPV-K would become dominant when high inoculum concentration was used (suggesting its presence as a minor component in the ChinNPV-H, -L, -N and -P dominant samples obtained in Section 2.4). For this, a total of 20 *C. includens* fifth instars were allowed to consume 1 × 10^8^ OBs/mL of each sample, which was expected to cause a 90–100% mortality. Inoculated larvae were reared individually in plastic cups until death or pupation. A group of 20 control larvae were treated identically, but did not consume OBs. The results were analyzed as described in Section 2.3, considering each larva as an individual replicate.

### 2.6. Prevalence of ChinNPV-K and ChinNPV-E REN Profiles in Larvae Inoculated with Mixtures of Variants K and E

In order to confirm the suspected concentration-diversity effect associated with the ChinNPV-K genotype, an experiment was designed to assess whether the ChinNPV-K genotype had to be present at a minimum concentration in the inoculum to generate variability, and if so, to determine the threshold concentration at which the ability of ChinNPV-K to generate that variability was lost. For this, we prepared different mixtures of ChinNPV-K OBs and ChinNPV-E OBs in a range of proportions maintaining a final concentration of 1 × 10^8^ OBs/mL, expected to result in >90% mortality (Table 1). A total of 20 fifth instar larvae were inoculated, reared individually, and those that died of polyhedrosis were individually subjected to OB extraction and REN analysis.

Additional tests were performed using variants K and E at a lower concentration of 5 × 10^4^ OBs/mL (Table 2), expected to result in 20–30% mortality. In this case, only three treatments were performed due to the low mortality that this concentration produces and the high number of insects that have to be inoculated to achieve a sufficient number of samples for analysis. For this, 150–200 larvae were inoculated in each treatment, reared individually, and those that died of polyhedrosis disease were subjected to OB extraction and REN analysis. In the case, treatments were applied across three different batches of larvae from the laboratory colony.

### 2.7. Prevalence of ChinNPV-K REN Profile in Pooled Samples

In all the previous experiments REN profiles were obtained from individual virus-killed larvae. The following experiment was performed to determine whether the ChinNPV-K REN profile could be detected when progeny OBs from virus-killed larvae that had been inoculated with a low concentration of ChinNPV-K OBs, were collected in a pooled sample. For this, 20 *C. includens* fifth instars were allowed to consume 5 × 10^4^ OBs/mL of ChinNPV-K pure variant using the droplet feeding method and were reared individually in plastic cups with diet. Larvae that died of polyhedrosis disease were collected, pooled and purified to produce a first passage inoculum. The purified inoculum was quantified by counting OBs and subjected to REN profile analysis.

We also examined whether the concentration diversity effect was maintained after using the pooled OB sample as inoculum. For this, three different batches of larvae were obtained from the laboratory colony and used for the following two treatments. Groups of 150–200 *C. includens* fifth instars were inoculated with the lower concentration (5 × 10^4^ OBs/mL) and 15–20 larvae were inoculated with the higher concentration (1 × 10^8^ OBs/mL) of the pooled OB inoculum, which was expected to cause between 20% and 99% mortality, respectively. Inoculated larvae were reared individually in plastic cups. Virus-killed larvae were recovered individually and each dead larva was processed and analyzed as described in Section 2.3. A total of approximately 100 larvae were analyzed for the low concentration experiment and 20 for the high concentration. Each larva was considered as a replicate.

### 2.8. Production of Budded Virus by ChinNPV-K and ChinNPV-E

The production of budded virus (BV) in larvae infected with ChinNPV-K and ChinNPV-E was determined by end point dilution assay. Briefly, 20 fifth instars of *C. includens* were inoculated with 10^8^ OBs/mL of each genotype by the droplet feeding method. Hemolymph was extracted from larvae by bleeding at 48 h post-infection (h.p.i.), pooled within each variant, and immediately frozen. The sample of hemolymph of each genotype was diluted 1:10 in TNM-FH medium (10^−1^), filtered through a 0.45 μm filter and serially diluted 1:10 in TNM-FH medium (10^−2^ to 10^−6^). For each variant, a 10 μL volume of each dilution was used to inoculate wells containing 10 ^4^ HighFive cells in a 96-well plate. A total of 24 wells were inoculated with each dilution and the experiment was performed three times (replicates). Plates were sealed with masking tape, incubated at 28 °C for 7 days and were then examined for signs of virus infection. Results were analyzed by the Spearman–Kärber method [22] in order to determine the 50% tissue culture infectious dose (TCID50) of each variant. The resulting values were compared by Welch’s *t*-test for unequal variances using SPSS v25.0 [21].

The quantity of BV of each genotype present in pooled hemolymph samples was then compared by qPCR using specific primers for ChinNPV in a CFX96 thermal cycler (Bio Rad, Hercules, CA, USA). For this, specific primers targeted at the ChinNPV *dnaPol* gene were used in a reaction prepared in 96-well optical plates and containing a mixture of 3 µL of SYBR Green Supermix (Bio Rad Laboratories, Hercules, CA, USA), 0.2 µL of each specific primer (10 µM), 5.6 µL of distilled water and 1 µL of template DNA, reaching a final volume of 10 µL. Genomic DNA of ChinNPV-K and -E pure variants was used as an internal standard for each qPCR assay. The amplification reaction protocol was: denaturation step at 95 °C for 2.3 min, 44 amplification cycles at 95 °C for 15 s and 60 °C for 30 s. The last step, a melting curve analysis, was performed with a dissociation stage of 60 °C for 15 s and 95 °C for 5 s, to confirm the presence of the target amplicon by the visualization of a single peak. Hemolymph samples of each variant were quantified in independent qPCR reactions. Posterior data analysis was performed using Bio-Rad CFX Manager software (Bio-Rad Laboratories, Hercules, CA, USA).

### 2.9. ChinNPV-K Replication in Cell Culture

To assess whether the behavior and productivity of the ChinNPV-K variant at high and low concentrations in cell culture were similar to that of the experiments performed in vivo, a series of experiments was performed in vitro.

#### 2.9.1. Cell Culture Infection at High BV Concentration

First, cells were inoculated with BVs at a high concentration. For this, 2 × 10^6^ HighFive cells were deposited in 25 cm^2^ flasks. After 1 h, cells attached to flask surface were inoculated with hemolymph collected from fifth instar larvae infected with ChinNPV-K or -E pure variants at a multiplicity of infection (MOI) of 10 to ensure synchronous infection of the majority of cells. As ChinNPV does not replicate rapidly, cells were recovered at 10 days post-infection and centrifuged at 3000× *g* for 10 min. Pelleted cells were subjected to DNA extraction following the MasterPure DNA Extraction Kit protocol (Epicentre Biotechnologies, Madison, WI, USA). Extracted DNA was analyzed by REN. This experiment was performed in triplicate.

#### 2.9.2. Cell Culture Infection at Low BV Concentration: Plaque Purification

As it was not possible to analyze REN profiles directly from cell cultures inoculated with a low concentration of BVs, due to the low production of virus progeny, plaque purification was performed and the recovered plaques were individually amplified in *C. includens* fifth instars. Samples of hemolymph from larvae infected with ChinNPV-K and -E pure variants were diluted in TNM-FH medium and then filtered through a 0.45 μm filter. Six-well tissue culture plates were seeded with 10^6^ cells/well and were inoculated with 200 μL of each dilution (10^−5^–10^−7^) of hemolymph samples. The plates were incubated for one hour at room temperature on an orbital shaker to allow virions to adsorb to the cells. Inoculum was then removed and a 2 mL layer of 2% SeaPlaque agarose (Lonza, Rockland, ME, USA) was added to the wells. Inoculated cell culture plates were incubated at 28 °C. After 10 days p.i., wells containing clearly separated plaques (i.e., wells with fewer than ten plaques) were selected for plaque picking and the individual plaques were collected with a sterile Pasteur pipette and diluted in 300 μL of TNM-FH medium. Individual plaque picks were then injected intrahemocelically in *C. includens* fifth instars that were placed individually in a plastic cup with semi-synthetic diet and incubated at 25 ± 1 °C and 75% relative humidity until death or pupation. Virus-killed larvae were collected individually, and the OBs were extracted, purified as described in Section 2.1 and subjected to REN analysis (Section 2.2). The prevalence of the recovered variants was expressed as the percentage of each variant’s REN profile observed in the total of virus-killed insects.

#### 2.9.3. Cell Culture Infection at Low ODV Concentration: Plaque Purification

Finally, an additional plaque purification procedure was performed using ODVs instead of BVs, in order to examine the possible differences between the two types of virions. Briefly, to release ODVs from the protein matrix, a 20 μL volume of ChinNPV-K or -E pure OB suspension (10^10^ OBs/mL) was incubated with 50 μL of 0.1 M Na_2_CO_3_ and 180 μL of distilled water at 28 °C for 30 min. This was performed three times. The suspension was centrifuged at 6000× *g* for 5 min and the supernatant containing ODVs was diluted in TNM FH medium and filtered through a 0.45 μm filter for use in plaque purification. The remaining steps of plaque purification and analysis were identical to those described in the study on BVs in hemolymph.

### 2.10. ChinNPV-K and ChinNPV-E Genome Sequencing

In order to determine whether differences in the ChinNPV-K and -E genome sequences could account for the biological differences observed, genomic DNA from ChinNPV-K and ChinNPV-E variants was extracted from purified OBs and its integrity was checked by gel electrophoresis. DNA samples were shipped to StabVida laboratories (Stab Vida Lda, Caparica, Portugal) for library construction and sequencing using an Illumina Miseq with 300 bp paired-end sequencing reads. Genome gap closing was performed using the Sanger technique after PCR amplification of the non-overlapping regions using specific primers. Next, de novo assembly was carried out using CLC Genomics Workbench 10.1.1 [23]. Raw sequence data were quality filtered by removing low quality or ambiguous reads that were <Q30, according to Illumina quality scores. Reads shorter than 50 bp were discarded. Reads were de novo assembled using a stringent criterion of overlap of at least 98 bp of the read and 98% identity. After contig creation, reads were mapped back to the contigs for assembly correction. The consensus sequences were digested in silico with BamHI, EcoRI, HindIII and PstI [24] to compare the REN profile patterns of the consensus sequence with those of the empirical observations. The raw sequences of each genotype were mapped against its consensus sequence to assess the intrinsic variability of both genomes using the variant detector tool of CLC [25]. Genome annotation for ChinNPV-K and ChinNPV-E consensus sequences was performed using SnapGene^®^ [26].

## 3. Results

### 3.1. Effects of Host Heterogeneity and Inoculum Concentration on Genetic Diversity Following Inoculation of Larvae with ChinNPV-Mex1

**Figure 1:**
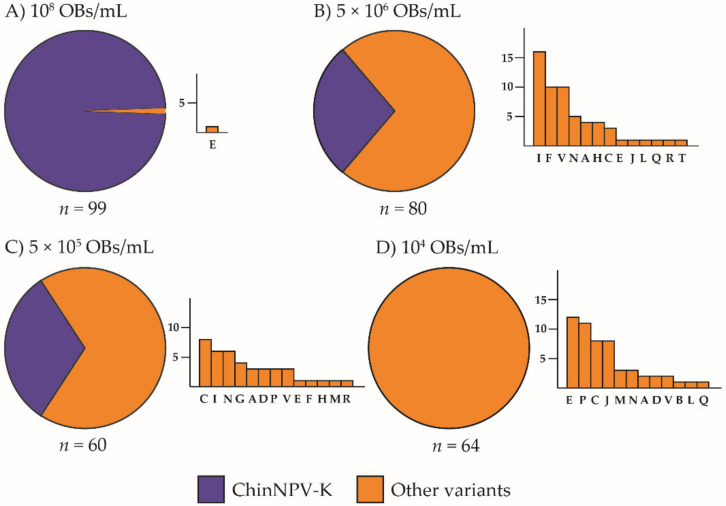
Prevalence of ChinNPV variants by dominant REN profile in *Chysodeixis includens* fifth instar larvae inoculated with ChinNPV-Mex1 OBs at concentrations of (**A**) 10^8^, (**B**) 5 × 10^6^, (**C**) 5 × 10^5^, (**D**) 10^4^ OBs/mL. Circular charts represent the proportions of dominant REN profiles similar or different to that of ChinNPV-K OBs recovered from virus-killed larvae (indicated as ChinNPV-K and Other variants, respectively). A bar chart is depicted next to each circular chart, specifying which REN profiles were observed among the other variants. The *y*-axis indicates the number of insects that showed a particular dominant REN profile in the progeny OBs and the *x*-axis indicates each of the REN profiles observed in descending frequency; *n* indicates the number of insects analyzed.

Virus-induced insect mortality varied significantly with inoculum concentration, from a minimum of 14% to a maximum of 100% at the lowest and highest concentrations, respectively. The frequency of the ChinNPV-K variant REN profile increased significantly with inoculum concentration (*χ*^2^ = 174.0, df = 3, *p* < 0.001). The high inoculum concentration (10^8^ OBs/mL) resulted in 100% mortality and 99 out of 100 recovered larvae showed the ChinNPV-K variant REN profile, whereas the single remaining insect had the dominant profile of the ChinNPV-E variant (Figure 1A).

The intermediate concentrations of 5 × 10^6^ and 5 × 10^5^ OBs/mL resulted in 76% and 40% mortality, respectively. These concentrations also resulted in intermediate prevalences of 32% and 28%, respectively, of the ChinNPV-K REN profile in OBs from virus-killed insects. At a concentration of 5 × 10^6^ OBs/mL, ChinNPV-I was the most prevalent variant followed by twelve other variants (Figure 1B). At 5 × 10^5^ OBs/mL, ChinNPV-C was the most prevalent of the minority variants (Figure 1C). The lowest inoculum concentration, 10^4^ OBs/mL, resulted in 14% mortality, but none of the virus-killed larvae produced a ChinNPV-K REN profile, whereas the ChinNPV E variant was the most prevalent REN profile, followed by eleven other variants (Figure 1D).

### 3.2. Genetic Diversity Present Following Inoculation with Variants ChinNPV-K and ChinNPV-E

**Figure 2:**
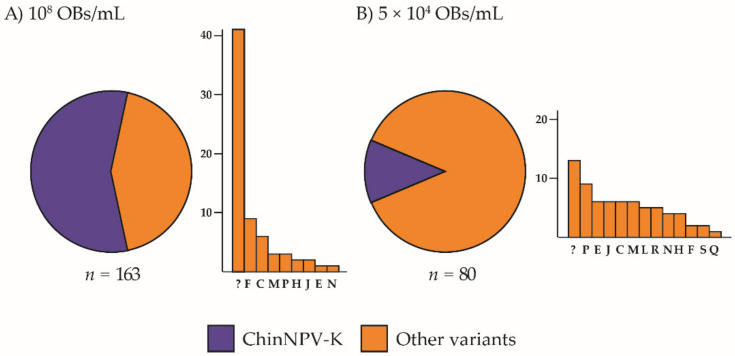
Prevalence of ChinNPV-K REN profiles in OB samples from virus-killed *Chrysodeixis includens* fifth instar larvae after inoculation with ChinNPV-K. Circular charts represent the proportions of REN profiles of ChinNPV-K and other variants in progeny OBs recovered from virus-killed larvae that had been inoculated with (**A**) 10^8^ OBs/mL or (**B**) 5 × 10^4^ OBs/mL. A bar chart is depicted next to each circular chart to indicate which REN profiles were recovered among the other variants. The *y*-axis indicates the number of insects that showed a particular dominant REN profile in the progeny OBs and the *x*-axis indicates each of the REN profiles observed in descending frequency; *n* indicates the number of insects analyzed. Novel REN profiles were classified as unknown and are shown as “?” in bar charts.

Inoculation of ChinNPV-K and ChinNPV-E pure variant OBs in *C. includens* fifth instars resulted in marked differences in the frequency of variants in virus-killed insects. Inoculum concentration significantly affected the prevalence of larvae showing the ChinNPV-K REN profile (*χ*^2^ = 41.84, df = 1, *p* < 0.001). The higher inoculum concentration of 10^8^ OBs/mL ChinNPV-K resulted in 99% mortality, but only 57% of larvae (90 out of 157 virus-killed insects) inoculated with the ChinNPV-K inoculum showed the ChinNPV-K variant REN profile (Figure 2A). The remaining 43% of insects showed one of nine different variants, the most prevalent of which was a previously unknown variant, present in 26% (41 out of 157 larvae) of virus-killed insects. The unknown variant (shown as “?” in Figure 2) did not match any of the variants identified previously [17]. The 5 × 10^4^ OBs/mL concentration of ChinNPV-K inoculum resulted in 22% mortality, and the prevalence of ChinNPV-K REN profiles in virus-killed insects was 13% (10 out of 79 insects). The remaining 87% of insects showed REN profiles of thirteen different variants, the most prevalent of which was a group of unknown variants present in 16% (13 out of 79 larvae) of virus-killed insects comprising REN profiles that did not match any of the variants identified previously [17].

In contrast, inoculum concentration did not significantly affect the prevalence of larvae showing the ChinNPV-E REN profile (*χ*^2^ = 1.62, df = 1, *p* = 0.579). When a high concentration of ChinNPV-E inoculum was used, 99% of larvae died of polyhedrosis disease and 97% (135 out of 139 larvae) of virus-killed insects showed the ChinNPV-E REN profile. A single unknown variant profile recovered from ChinNPV-E inoculated insects did not match any of the known variants. Larvae inoculated with the low concentration of ChinNPV-E inoculum experienced 24% mortality and the ChinNPV-E variant REN profile was the only profile observed in these insects. None of the control larvae died of polyhedrosis disease in any treatment.

### 3.3. Genetic Stability of Progeny OBs Obtained Following Infection with ChinNPV-K OBs at Low Concentration

Following the inoculation of larvae with a low concentration (5 × 10^4^ OBs/mL) of ChinNPV-K variant OBs in the previous experiment (Section 3.2), we selected four samples of the resulting variants showing the REN profiles of ChinNPV-H, -L, -N and P. These samples were then used to inoculate larvae at high OB concentration (10^8^ OBs/mL) to see whether it was possible to recover the original ChinNPV-K REN profile. Analysis of OBs from virus-killed larvae revealed that all four samples tested conserved their original REN profile (i.e., ChinNPV-H, -L, -N and -P), and no evidence of the ChinNPV-K variant or any other variant was detected in any of the virus-killed insects. This suggests that for the ChinNPV-K variant to dominate in the OB progeny, it must be present at a minimum concentration in the OB inoculum administered to larvae under high concentration conditions.

### 3.4. Prevalence of ChinNPV-K and ChinNPV-E REN Profiles in Larvae Inoculated with Mixtures of Variants K and E

**Figure 3:**
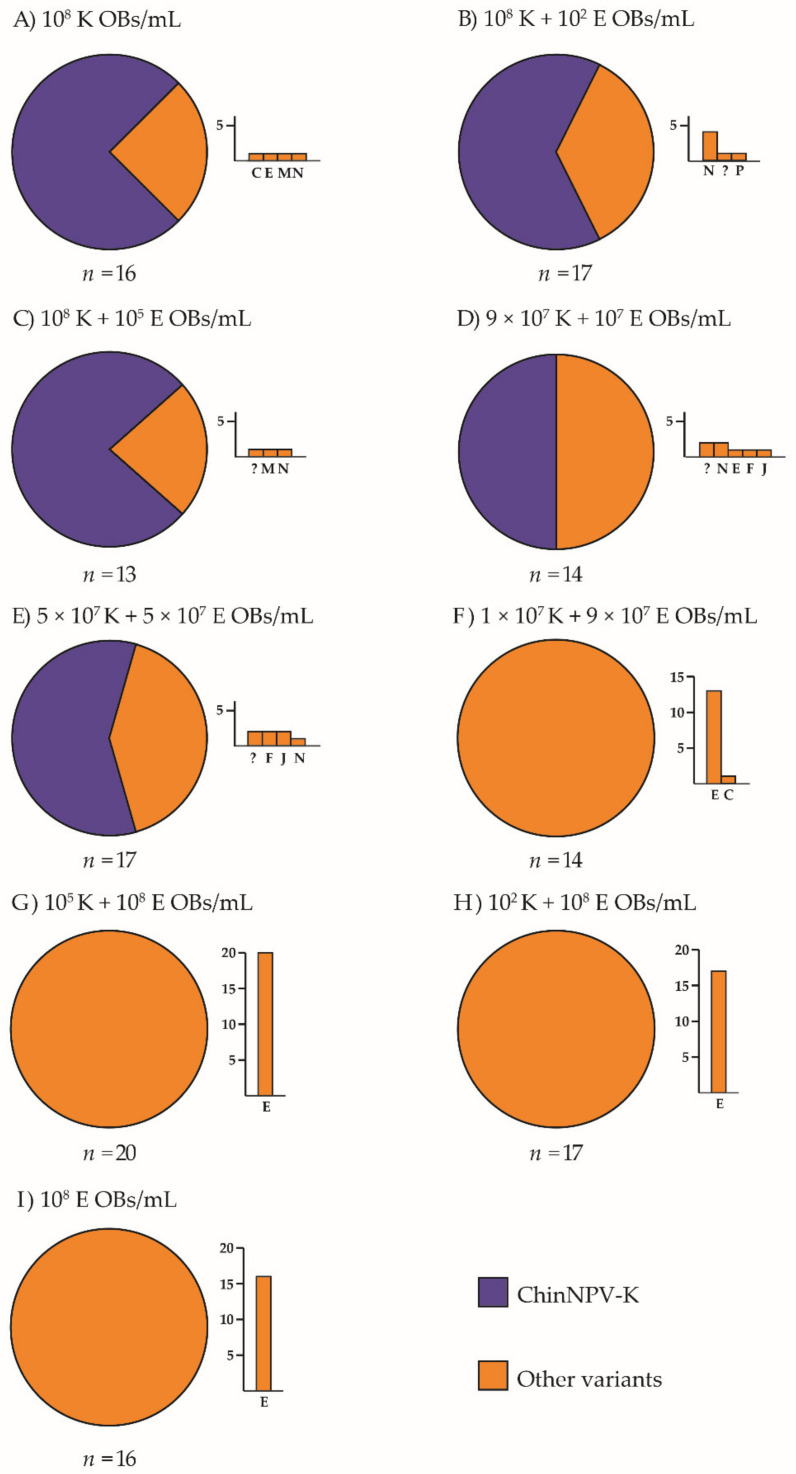
Prevalence of REN profiles in *Chrysodeixis includens* fifth instar larvae that died following inoculation of mixtures of variant K OBs and variant E OBs at high inoculum concentration (total concentration 10^8^ OBs/mL). Inocula were (**A**) 1 × 10^8^ K, (**B**) 10^8^ K + 10^2^ E, (**C**) 10^8^ K + 10^5^ E, (**D**) 9 × 10^7^ K + 10^7^ E, (**E**) 5 × 10^7^ K + 5 × 10^7^ E OBs/mL, (**F**) 1 × 10^7^ K + 9 × 10^7^ E OBs/mL, (**G**) 10^5^ K + 10^8^ E OBs/mL, (**H**) 10^2^ K + 10^8^ E OBs/mL and (**I**) 10^8^ E OBs/mL. Circular charts represent the proportions of REN profiles of ChinNPV-K and other variants recovered from dead larvae. A bar chart depicted next to each circular chart indicates which REN profiles were recovered among the other variants and their frequency. The *y*-axis indicates the number of insects that showed a particular dominant REN profile in the progeny OBs and the *x*-axis indicates each of the REN profiles observed in descending frequency; *n* indicates the number of insects analyzed. Novel REN profiles were classified as unknown and are shown as “?” in bar charts.

The mixtures comprising similar or higher proportions of ChinNPV-K than ChinNPV-E revealed that ChinNPV-K REN profiles were observed in the OB progeny from the majority of insects that were inoculated with a high OB concentration. This was so even in the treatment in which the inoculum comprised both variants in equal proportions (5 × 10^7^ OBs/mL of ChinNPV-K + 5 × 10^7^ OBs/mL of ChinNPV-E) (Figure 3).

Other variant REN profiles (variants C, E, F, J, M, N, P and one new variant, not classified previously) appeared in mixtures in which ChinNPV-K was present in the inoculum in a similar or higher proportion than ChinNPV-E. When ChinNPV-E was present in the inoculum at a higher concentration than ChinNPV-K, only the ChinNPV-E REN profile was observed in OBs from virus-killed insects, except for one case (variant ChinNPV-C) recovered in the mixture in which ChinNPV-E OBs were nine-fold more abundant than ChinNPV-K OBs (1 × 10^7^ K + 9 × 10^7^ E OBs/mL).

**Figure 4:**
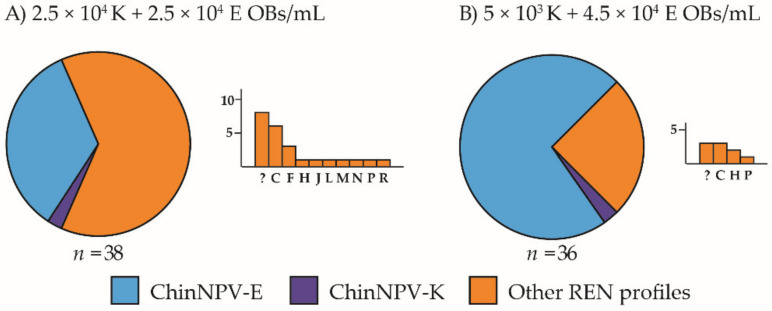
Prevalence of ChinNPV-K and ChinNPV-E REN profiles in *Chrysodeixis includens* fifth instar larvae that died following inoculation with variant mixtures (K + E) at low inoculum concentration (total concentration 5 × 10^4^ OBs/mL). Inocula were (**A**) 2.5 × 10^4^ K + 2.5 × 10^4^ E OBs/mL, (**B**) 5 × 10^3^ K + 4.5 × 10^4^ E OBs/mL. Circular charts represent the proportions of REN profiles of ChinNPV-E, ChinNPV-K and other variants recovered from dead larvae. A bar chart depicted next to each circular chart indicates which REN profiles were recovered among the other variants and their frequency. The *y*-axis indicates the number of insects that showed a particular dominant REN profile in the progeny OBs and the *x*-axis indicates each of the REN profiles observed in descending frequency; *n* indicates the number of insects analyzed. Novel REN profiles were classified as unknown and are shown as “?” in bar charts.

For the lower concentration, 5 × 10^4^ OBs/mL, in both inoculum mixtures that contained the ChinNPV-K genotype (2.5 × 10^4^ K + 2.5 × 10^4^ E and 5 × 10^3^ K + 4.5 × 10^4^ E OBs/mL), the K variant REN profile appeared only once in each treatment. In the equimolar mixture (2.5 × 10^4^ K + 2.5 × 10^4^ E OBs/mL), 34% (13 out of 38 insects) of the recovered REN profiles showed the ChinNPV-E REN profile and up to 10 different REN profiles were detected in virus-killed insects (Figure 4A). In the inoculum in which the concentration of ChinNPV-K OBs was ten-fold higher than that of the ChinNPV-E OBs (5 × 10^3^ K + 4.5 × 10^4^ E OBs/mL), the ChinNPV-E REN profile was observed in 72% of virus-killed insects, with only four different variant REN profiles among the other REN profiles observed. In contrast, the diversity of different variant profiles decreased with the increasing concentration of ChinNPV-K OBs in the inoculum, with only five variants observed (apart from ChinNPV-K), most of which were of ChinNPV-E (Figure 4B). In the inoculum comprising ChinNPV-E OBs alone, no other variant profiles were recovered (not shown in Figure 4). These results indicate again that ChinNPV-E behaved as a pure genotype and was not involved in the spontaneous generation of ChinNPV diversity.

### 3.5. Prevalence of ChinNPV-K REN Profile in Pooled Samples

**Figure 5:**
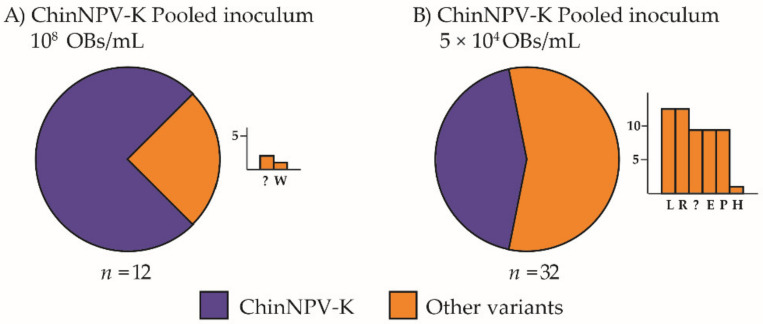
Prevalence of REN profiles in *Chrysodeixis includens* fifth instar larvae that died following inoculation with (**A**) 10^8^ OBs/mL or (**B**) 5 × 10^4^ OBs/mL of a pooled OB inoculum derived from insects that had been originally inoculated with ChinNPV-K variant OBs. Circular charts represent the proportion of REN profiles of ChinNPV-K and other variants recovered from dead larvae. A bar chart is depicted next to each circular chart specifying which REN profiles were recovered among the other variant profiles and their frequency. The *y*-axis indicates the number of insects that showed a particular dominant REN profile in the progeny OBs and the *x*-axis indicates each of the REN profiles observed in descending frequency; *n* indicates the number of insects analyzed. Novel REN profiles were classified as unknown and are shown as “?” in bar charts.

The OB samples collected following the inoculation of larvae with ChinNPV-K at the lower concentration (5 × 10^4^ OBs/mL) were pooled and used to inoculate new batches of larvae at two inoculum concentrations to determine whether the ChinNPV-K variant could be recovered by a single passage of pooled OB samples. The REN profile of the pooled OB sample was identical to that of the ChinNPV-K variant profile. When pooled OBs were used to inoculate larvae at the higher concentration (1 × 10^8^ OBs/mL), 9 out of 12 virus-killed larvae (75%) presented the ChinNPV-K variant profile, whereas the remaining 3 larvae (25%) presented two different variant profiles (Figure 5A). When pooled OBs were used to inoculate larvae at the lower concentration (5 × 10^4^ OBs/mL), the ChinNPV-K variant was again the most prevalent profile present in 14 out of 32 larvae (44%) of the total recovered genotypes. The remaining 18 insects (56%) presented one of six different variant profiles in different frequencies (Figure 5B). These results suggest that: (i) pooling the OBs of multiple variants may result in a variant K-type consensus REN profile and (ii) that the pooled OBs behaved similarly to pure ChinNPV-K variant inoculum in that the ChinNPV-K variant profile predominated in most larvae that died from high concentration pooled inoculum, whereas considerable diversity was observed in larvae that died from low concentration pooled inoculum.

### 3.6. Production of Budded Virus by ChinNPV-K and ChinNPV-E

TCID_50_ values estimated by end-point dilution assay were 1.3 × 10^6^ ± 2.3 × 10^5^ pfu/mL for ChinNPV-K and 2.8 × 10^5^ ± 1.9 × 10^4^ pfu/mL for ChinNPV-E, indicating a significantly higher production of BV in the hemolymph of ChinNPV-K infected larvae at 48 h post-infection (Welch’s *t* = 19.88, df = 1, 2.03, *p* = 0.046). Budded virus quantification by qPCR indicated the presence of 1.95 × 10^8^ genome copies/mL of hemolymph for ChinNPV-K and 8.5 × 10^7^ copies/mL for ChinNPV-E, i.e., a 2.3-fold higher production of BV in ChinNPV-K infected insects.

### 3.7. ChinNPV-K Replication in Cell Culture

#### 3.7.1. Cell Culture Infection at High BV Concentration

The infection of HighFive cells with a high concentration of ChinNPV-K BVs resulted in a DNA profile that matched that of the ChinNPV-K variant in each of the three replicates performed.

#### 3.7.2. Cell Culture Infection at Low BV Concentration: Plaque Purification

**Figure 6:**
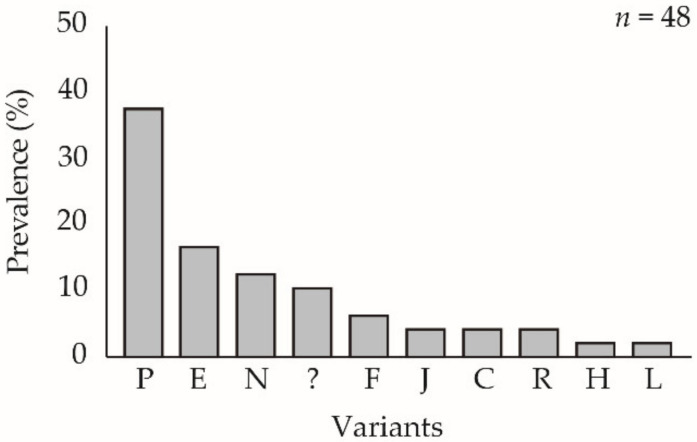
Prevalence of REN profiles from virus-killed larvae that were infected by injection of plaque picks derived from BVs in hemolymph samples obtained following inoculation of *Chrysodeixis includens* fifth instars with ChinNPV-K pure variant OBs. New genotypes whose REN profiles did not match any of the previous studied variants were included in an unknown group shown as “?”; *n* indicates the number of plaque picks used to inoculate insects. The *y*-axis indicates the prevalence (%) of insects that produced progeny OBs with a particular dominant REN profile and the *x*-axis indicates each of the observed REN profiles in decreasing frequency.

For the lower concentration, a total of 48 plaque picks that originated from ChinNPV-K BVs in hemolymph were injected in *C. includens* fifth instars and virus-killed larvae that produced OBs were analyzed by REN. Ten different REN profiles were identified in virus-killed insects, but none of the 48 clones presented the characteristic ChinNPV-K variant REN profile (Figure 6). The most prevalent REN profile in cell culture was that of ChinNPV-P that was present in 18 out of 48 (38%) virus-killed larvae. In contrast, plaque picks derived from ChinNPV-E BV in hemolymph, produced virus-killed larvae that showed the ChinNPV E variant profile alone, and no other REN profiles were observed. Thus, ChinNPV-E again behaved as a pure genetic variant and did not spontaneously generate variant diversity.

#### 3.7.3. Cell Culture Infection at Low ODV Concentration: Plaque Purification

**Figure 7:**
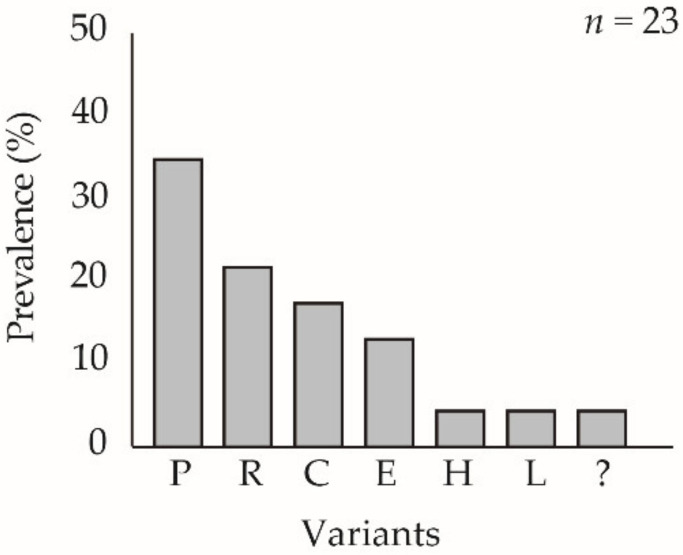
Prevalence of REN profiles from virus-killed *Chrysodeixis includens* fifth instars that died following injection of plaque picks derived from ChinNPV-K pure variant ODVs. New genotypes whose REN profiles did not match any of the previous studied variants were included in an unknown group shown as “?”; *n* indicates the number of plaque picks used to inoculate insects. The *y*-axis indicates the prevalence (%) of insects that produced progeny OBs with a particular dominant REN profile and the *x*-axis indicates each of the observed REN profiles in decreasing frequency.

The previous plaque purification procedure was repeated using ChinNPV-K ODVs instead of BVs, with similar results. Seven different REN profiles from 23 analyzed plaque picks were observed in virus-killed insects, but none of these corresponded to the ChinNPV-K variant profile (Figure 7). The genetic variants obtained from the plaque picks using ODVs as inoculum differed from those obtained using BV in hemolymph as inoculum, although variant P was the dominant variant profile in both cases. This result suggests that the ChinNPV-K variant is not a mixture of genetic variants but is capable of generating variant diversity de novo. It also indicates that the host system (larva or cell culture) did not influence the generation of diversity, as broadly similar levels of variant diversity were observed in both systems, although variants differed in prevalence in each system.

### 3.8. ChinNPV-K and ChinNPV-E Genome Sequencing

**Figure 8:**
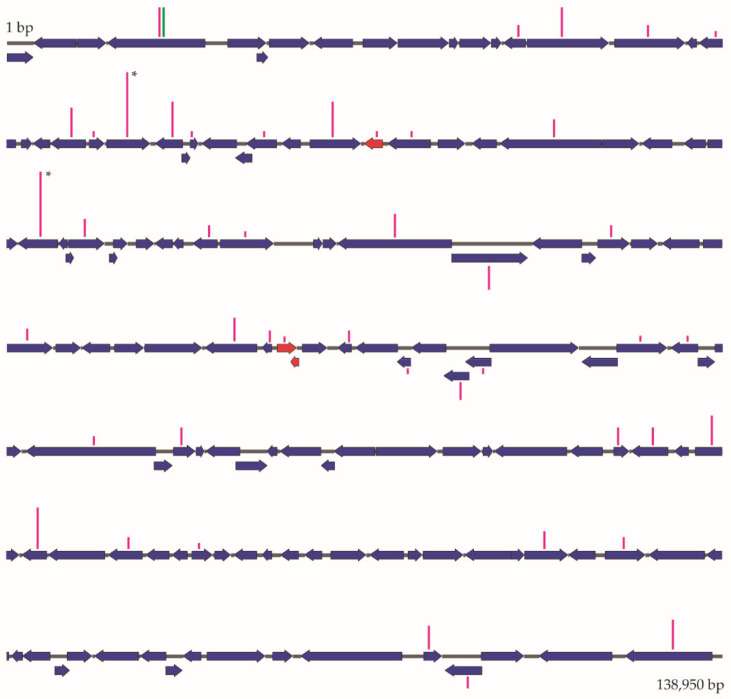
Schematic representation of ChinNPV-K genome ORFs. Vertical bars represent the number of cumulative SNPs in each ORF in pink for ChinNPV-K and green for ChinNPV-E, resulting in different heights according to the number of SNPs. Bars labelled with asterisks indicate the highest number of SNPs detected, which was 22 SNPs per ORF. Only the ChinNPV-K genome is depicted due to the high similarity between the ChinNPV-K and ChinNPV-E ORF length and distribution. The three ORFs in red represent ORFs that are not present in the ChinNPV genomes sequenced previously.

Raw sequence data of both variants gave an entire coverage of 6291 for ChinNPV-E and 6180 for ChinNPV-K. Reads obtained by Illumina technology for ChinNPV-E DNA were assembled in a unique contig, whereas the sequence of ChinNPV-K was assembled in six different contigs and subsequently closed by Sanger sequencing. For both sequences, the Phred quality score was >30. ChinNPV-K and ChinNPV-E consensus sequences were digested in silico. The ChinNPV-E in silico REN profile with EcoRI and HindIII both exactly matched the empirical profiles, whereas the ChinNPV-K in silico REN profile did not match either the EcoRI or the HindIII empirical profiles, although it did match those of the other restriction enzymes tested, such as PstI and BamHI. After mapping the raw reads of both ChinNPV-E and ChinNPV-K against its own consensus sequence, ChinNPV-E showed a small degree of variation located in the *hoar* gene, which did not include any restriction site. However, the ChinNPV-K sequences showed variability along the entire genome (Figure 8). Indeed, 394 variations were detected in ChinNPV-K after Illumina sequencing (Supplementary Table S1), most of which were SNPs, indicating a mutation rate of 2 × 10^−3^ substitutions per nucleotide, compared to 6.4 × 10^−5^ substitutions/nt in ChinNPV-E (based on 9 SNPs). Four of the SNPs in the ChinNPV-K genome were located in EcoRI restriction sites, which may explain why the in silico and the experimental REN profiles did not match. Genome annotation using the Pseudoplusia includens SNPV-IE reference genome, resulted in 139 ORFs for both genomes (two fewer than the PsinSNPV reference genome), of which three ORFs had no correspondence with any of the sequenced ChinNPV genomes available in the NCBI GenBank. Differences between ChinNPV-K and -E genomes were mainly SNPs, which did not affect the length of any of the detected ORFs, with one marked exception in the *hoar* gene, which was 114 bp longer in ChinNPV-E compared to the PsinSNPV reference genome.

## 4. Discussion

In this study we examined characteristics of OBs collected from insects inoculated with different concentrations of ChinNPV-K and ChinNPV-E OBs. In larvae inoculated with the K variant the number of genotypic variants present in individual virus-killed insects increased as the concentration of inoculum decreased. Genome sequencing revealed that the ChinNPV-K variant harbored a high level of intrinsic genetic variability that could be readily detected by the REN technique. The unstable ChinNPV-K variant was capable of generating a range of genotypes that appeared on multiple occasions and at varying prevalence in insects inoculated with low concentrations of ChinNPV-K OBs. The appearance of so many variants and the differences in the abundance of ChinNPV-K in virus-killed insects from high and low inoculum concentrations indicated that ChinNPV-K was not a structured mixture of genotypes. Indeed, considering that both genotypes (ChinNPV-K and ChinNPV-E) were obtained through plaque purification, it is highly unlikely that all the genotypes produced in ChinNPV-K killed insects were collected along with the ChinNPV-K variant when this genotype was isolated by plaque purification.

These results find clear support from previous studies on Helicoverpa armigera nucleopolyhedrovirus (HearNPV) [16], in which high variability of HearNPV was observed in insects inoculated with low doses of OBs. Nevertheless, there are important differences with the procedures of Baillie and Bouwer [16] in that the HearNPV study was performed using two isolates, whereas we used a mixture of nine different field isolates of ChinNPV, i.e., our initial inoculum was likely to comprise a greater diversity of variants than the inoculum used by Baillie and Bouwer [16].

Baillie and Bouwer [16] argued that genetic events like mutation and recombination are expected to occur at similar rates under both high and low dose conditions, although a lower production of OBs in the insects that consumed high dose inoculum (due to the fast speed-of-kill associated with high inoculum concentrations) may reduce the genetic diversity detected. Recombination events take place when the same cell is infected by at least two different genotypes and this may be exacerbated at high multiplicities of infection [27], which would result in higher variability when large quantities of virus are used as inoculum. However, we observed the contrary, namely greater diversity at low inoculum concentrations.

This may be explained by a founder effect, in which a few founder genomes undergo changes in the early stages of infection which are then transmitted and amplified in other cells via the BV progeny and become the dominant variant in the majority of OB progeny collected from virus-killed insects. This effect relies on a combination of (i) the paucity of founder virus genomes in insects that consume small quantities of inoculum, as the infection of midgut cells represents an important bottleneck to the transmission of diversity [28,29], (ii) instability in the replication of these genomes early in infection and (iii) stochastic events generated by host genotype or immune condition that determine variant survival. Such effects are likely to be most evident when the founder virus population size is small [29,30]. Intuitively, we would predict the influence of founder effects on variant diversity to be potentiated when variants also differ in their rate of replication. This is because rare but fast replicating variants have fewer replication cycles in which to increase in prevalence if the host dies prematurely. The relationship between the speed-of-kill of the virus and the quantity of OBs consumed has been recognized often in alphabaculovirus pathosystems [14,15,28]. Although we compared only two variants, it was clear that variant K was faster in replication than variant E with a 4.6-fold (end-point dilution estimate) or 2.3-fold (qPCR estimate) higher BV production by variant K at 48 h post-infection and one of the highest levels of OB production of all of the variants analyzed in a previous study [17]. Notably, of all the variants that we identified, instability was only observed in the ChinNPV-K variant, whereas all other variants were stable during passage in *C. includens*.

The diversity of infection in an insect is also likely to be modulated by the phenomenon of superinfection exclusion. Superinfection exclusion is employed by many viruses, including baculoviruses, to avoid coinfection of cells by similar viruses [31,32,33]. Recently, this been proposed as a mechanism to limit co-infection of cells by parental viruses and their progeny that may harbor deleterious mutations [34]. As such, the greatest contribution to intrahost diversity is likely to originate from the progeny of the founder variants that disperse as BVs during cycles of replication early in infection, whereas superinfection exclusion would restrict the proliferation of additional diversity later in infection when many cells are already infected. This idea finds some empirical support from a study in which the relative abundance of variants in *S. frugiperda* larvae infected by a wild-type isolate of SfMNPV did not change significantly during the entire period in which larvae died of polyhedrosis disease (67–139 h post-infection), although diversity at the initial stages of infection was not examined [35].

A minimum quantity of ChinNPV-K was required in the inoculum in order to observe the dose-dependent generation of variability. At high concentrations, a minimum of 50% of ChinNPV-K OBs was needed to observe the dominance of the ChinNPV-K variant in REN profiles, whereas the generation of diversity at low concentrations was observed even when only 10% of ChinNPV-K OBs was present in the inoculum. This finding confirmed that the ChinNPV-K variant functions as a generator of variability in an inverse concentration dependent manner.

At high inoculum concentrations, the ChinNPV-K genotype dominated all other variants in progeny OBs and, at the same time, the ChinNPV-E variant increased in prevalence at the intermediate inoculum concentrations (5 × 10^6^ and 5 × 10^5^ OBs/mL), and was the most prevalent variant at the lowest inoculum concentration. In contrast, Baillie and Bouwer [16] did not observe any consistent pattern in the distribution of genetic variants across inoculum doses in HearNPV. It has been hypothesized that when one genetic variant is particularly virulent, it can dominate over the other variants during the spread of an epizootic of infection [36]. This is usually observed in wild-type baculoviruses where the most abundant variant is often the one with the highest fitness in a given environment at a given moment [37,38]. Virus epizootics, however, involve selection during between-host transmission followed by within-host selection for a different set of traits [29]. Nevertheless, according to our previous observations [17], the ChinNPV-K genotype was not the most pathogenic variant i.e., the variant that produced the highest prevalence of mortality in inoculated insects. Indeed, ChinNPV-K was among the least pathogenic variants and resulted in mortality similar to that of the ChinNPV-Mex1 mixture [17]. This again emphasizes that these viruses are transmitted as groups of variants, the interactions among which determine their overall fitness [15].

To identify any genetic basis for the replication instability of ChinNPV-K, genome sequencing was performed and compared to the stable E variant. The ChinNPV-E reads, assembled in a single contig, showed little variability compared to the consensus sequence (with only 9 SNPs detected) and that variability was exclusively located in the *hoar* gene, which is known as a hypervariable region among baculovirus genotypes [3,39,40,41]. Deletion of *hoar* resulted in a marked reduction in OB pathogenicity in a SeMNPV bacmid and may be involved in the ODV infection of midgut cells [42,43]. In contrast, the ChinNPV-K reads had to be assembled in six contigs and had up to 394 variations in comparison to the consensus sequence that were distributed throughout the genome. The mutation rate for ChinNPV-K (2 × 10^−3^ substitutions/nt) was similar to that of another NPV (~1 × 10^−3^ substitutions/nt [12]), but higher than for other dsDNA viruses (~10^−6^ substitutions/nt) [2]. Furthermore, the presence of these SNPs at restriction sites is likely to be the origin of some of the variability observed. When one of these SNPs is fixed, the REN profile changes and, therefore, the resulting progeny OBs are considered as a new genetic variant. Most SNPs would not have biological relevance unless they produced a significant change in an amino acid at the active site of a particular protein, or a key cellular receptor, or in a critical promoter region of an essential gene or an important auxiliary gene.

It appears likely, therefore, that variants were generated during replication in cell culture, principally by point mutation. Recent studies in different baculoviruses using deep sequencing seem to indicate that point mutations are a frequent source of diversity in this virus family [12,25,29,36]. Therefore, genetic variability within natural baculovirus isolates is probably far higher than that observed with techniques, such as REN profiles or the DGGE (denaturing gradient gel electrophoresis) analysis used by Baillie and Bouwer [16].

Comparison of the ChinNPV-E and -K genomes did not reveal evidence for large deletions or insertions observed in variants from some other baculovirus populations [44,45,46], although a subset of ChinNPV isolates was recently identified comprising chimeric genomes that appear to survive in natural populations of this virus [47]. The pattern of variation in the ChinNPV-K genome did not match the regions of phylogenetic incongruence (PIRs) that have been described in Brazilian isolates of ChinNPV [47], in which variability was grouped across six defined zones of the genome, meaning that mechanisms other than horizontal gene transfer are responsible for the variability observed in ChinNPV-K.

The replication instability of ChinNPV-K also complicated potential further studies, such as the quantification of ChinNPV-K present in individual larvae by qPCR. Specifically, the unique primer targets for ChinNPV-K are likely to be compromised by SNPs or involve the *hoar* region that comprises numerous repeat sequences that invalidate its use in qPCR amplification. Consequently, despite its evident limitations, the use of restriction endonucleases was one of the few informative techniques available to us for variant characterization in the present study.

Although understanding the mechanism and fitness consequences of ChinNPV variant diversity were outside the scope of our study, our findings call into question the how and why of the replication instability of the K variant. Of the 394 SNPs detected in the K variant genome, 23 were located in genes directly involved in genome replication (*dna pol*, *helicase*, *alkaline exonuclease*). An additional 43 SNPs were located in genes encoding proteins possibly involved in genome replication, several of which have DNA binding activity (*39 k*/*pp31*, *pcna*, *bro-a*, *bro-b*, *ie-1*, *orf23*) (Supplementary Table S1). However, the mechanism for generating diversity in variant K is presently unclear.

Why would high levels of replication infidelity be beneficial? Genotypic diversity is selectively advantageous and has an entire set of fitness benefits to NPVs. Mixtures of variants can cooperate to increase the virus’ ability to establish a lethal infection and increase the total production of OBs (reviewed in [15]). Variant heterogeneity can also provide preadaptation that allows the virus to exploit a range of host genotypes [48,49,50], or improve their pathogenicity when host larvae feed on different species of food plants [51,52], or if alternative host species are present [53,54]. In addition, variant diversity can be advantageous when opportunities for vertical or horizontal transmission fluctuate in response to changes in host density [55], or when some variants lack transmission-modulating genes [15]. Critically, the benefits of variant diversity are possible because variants are not transmitted alone, but as genotypically diverse OBs and ODVs [56] that can be considered as collective infectious units [57]. This permits the transmission and intergenerational persistence of rare variants and even defective variants that can survive in the virus population, even when larvae consume low doses of inoculum [56,58]. Under such circumstances, the unit of selection during transmission is not the variant genome within an ODV, but the group of co-occluded variants.

In the case of the ChinNPV-Mex isolate, the presence of the K variant likely confers an advantage on the virus population as a means to overcome host and environmental variation. The K variant was dominant at high inoculum doses, a situation in which the transmission of minority variants is also assured by co-occlusion within OBs, whereas the same variant acts as a variability generator when the quantity of inoculum is low, and the number of founder variants is reduced. As such, the prevalence of the K variant in the wild-type isolate likely reflects a trade-off between its capacity to generate novel variants to overcome host and environmental variability and the increased likelihood of producing numerous structural variants with reduced fitness [13], or even defective variants that can only survive through complementation with viable genotypes [45,59]. As the number of studies on the genetic structure of NPV populations continues to grow, we may expect to see additional examples of virus populations that harbor replication unstable variants that contribute to population survival.

## 5. Conclusions

The ability of the ChinNPV-K variant to generate diversity likely represents one mechanism by which adaptive variation is introduced into hosts that consume low levels of virus inoculum. A combination of small founder populations in insects that consume few OBs, stochastic events and differences in variant replication rates is likely to modulate intra-host diversity that is usually transmitted in groups in genotypically diverse OBs. Genome sequencing revealed high sequence variability present in the in form of SNPs in the ChinNPV-K genome compared to limited variation present in the stably replicating ChinNPV-E variant. We conclude that ChinNPV-K is an unusual generator of variability in the natural Mexican isolates of ChinNPV that we examined.

## Figures and Tables

**Table 1:** Concentrations of ChinNPV-K and ChinNPV-E in inoculum mixtures with a total concentration of 10^8^ OBs/mL.

Treatment	ChinNPV-K	ChinNPV-E
Control	0	0
1	10^8^	0
2	10^8^	10^2^
3	10^8^	10^5^
4	9 × 10^7^	10^7^
5	5 × 10^7^	5 × 10^7^
6	10^7^	9 × 10^7^
7	10^5^	10^8^
8	10^2^	10^8^
9	0	10^8^

**Table 2:** Concentrations of ChinNPV-K and ChinNPV-E in inoculum mixtures with a total concentration of 5 × 10^4^ OBs/mL.

Treatment	ChinNPV-K	ChinNPV-E
Control	0	0
1	2.5 × 10^4^	2.5 × 10^4^
2	5 × 10^3^	4.5 × 10^4^
3	0	5 × 10^4^

## Data Availability

The data presented in this study are available in Figure 1, Figure 2, Figure 3, Figure 4, Figure 5, Figure 6, Figure 7 and Supplementary Table S1.

## Supplementary Materials

The following are available online at https://www.mdpi.com/article/10.3390/v13101895/s1, Table S1: ChinNPV-K_SNPs.
